# Supplementary material for: First approach to the population structure of Mycobacterium tuberculosis complex in the indigenous population in Puerto Nariño-Amazonas, Colombia
Source: PLoS One. 2021 Jan 7;16(1):e0245084. doi: 10.1371/journal.pone.0245084 (PMC7790298; doi:10.1371/journal.pone.0245084)
Supplement: S3 Fig — TB cases are indicated according to the genotypes found. Red: cluster 1; light blue: cluster 3; brown: orphan pattern. Scale bars indicate distance in meters. Reprinted from OpenStreetMap and QGIS 2.18.9 Las Palmas under a CC BY-SA 2.0 license. (PDF) [file pone.0245084.s004.pdf]

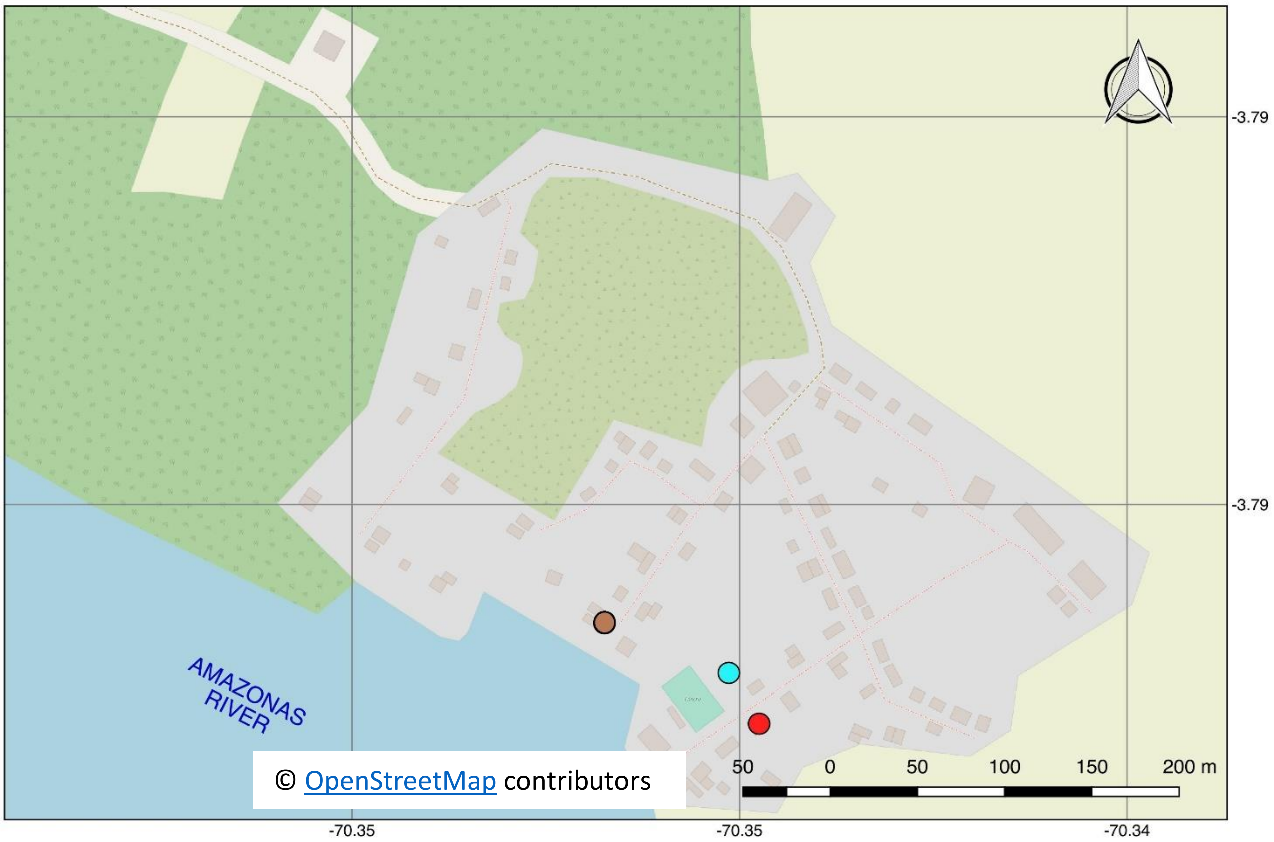

S3 Fig. Puerto Esperanza. TB cases are indicated according to the genotypes found. Red: cluster 1; light blue: cluster 3; brown: orphan pattern. Scale bars indicate distance in meters. Reprinted from OpenStreetMap and QGIS 2.18.9 Las Palmas under a CC BY-SA 2.0 license.
