# Supplementary figures and images for: First approach to the population structure of Mycobacterium tuberculosis complex in the indigenous population in Puerto Nariño-Amazonas, Colombia
Source: PLoS One. 2021 Jan 7;16(1):e0245084. doi: 10.1371/journal.pone.0245084 (PMC7790298; doi:10.1371/journal.pone.0245084)

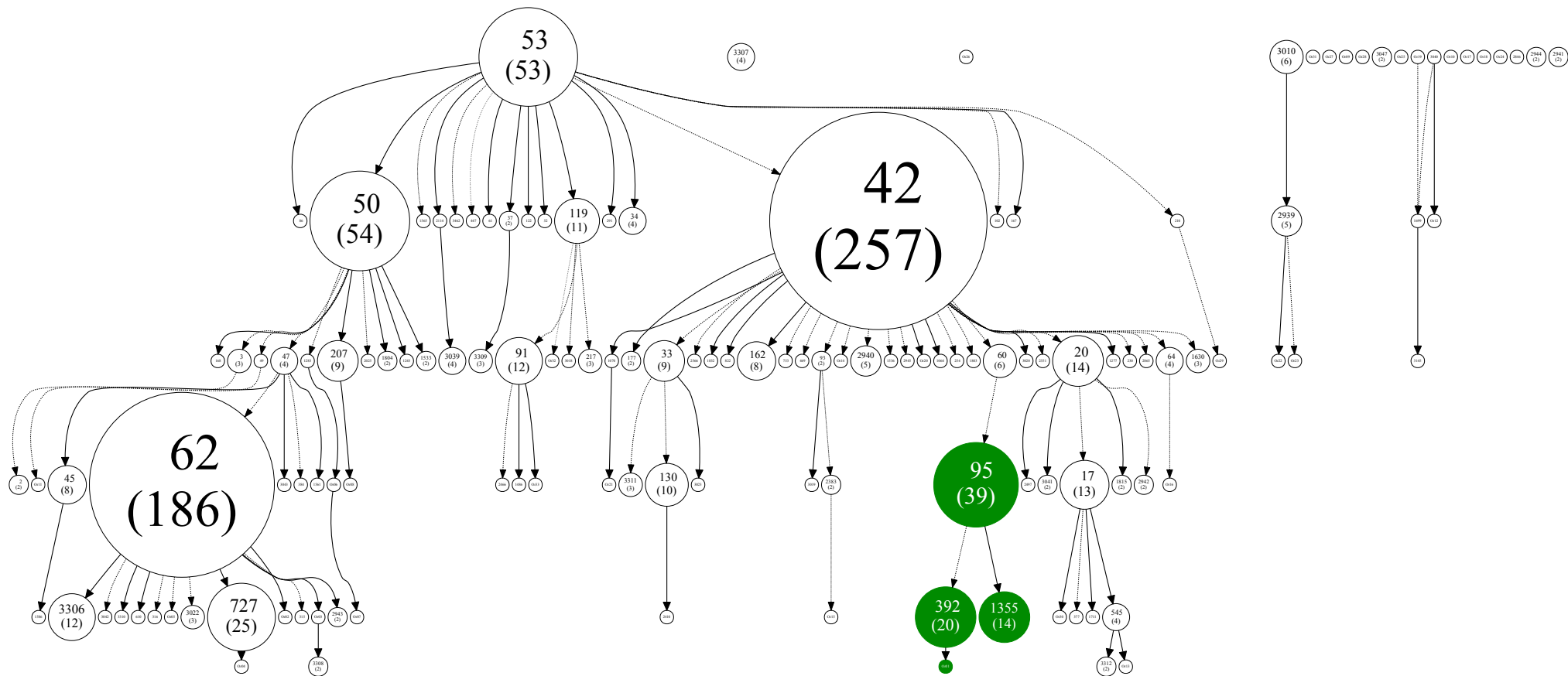

Supplement: S12 Fig — Each spoligotype pattern from the study is represented by a node with area size being proportional to the total number of isolates with that specific pattern (number shown in brackets under the SIT number). Changes (loss of spacers) are represented by directed edges between nodes, with the arrowheads pointing to descendant spoligotypes. Nodes corresponding to spoligotypes from this study (focusing on Indigenous population) were colored in green. (PDF) [file pone.0245084.s013.pdf]
