# Supplementary material for: First approach to the population structure of Mycobacterium tuberculosis complex in the indigenous population in Puerto Nariño-Amazonas, Colombia
Source: PLoS One. 2021 Jan 7;16(1):e0245084. doi: 10.1371/journal.pone.0245084 (PMC7790298; doi:10.1371/journal.pone.0245084)

UPGMA-Tree, MIRU-VNTR [24]: Categorical

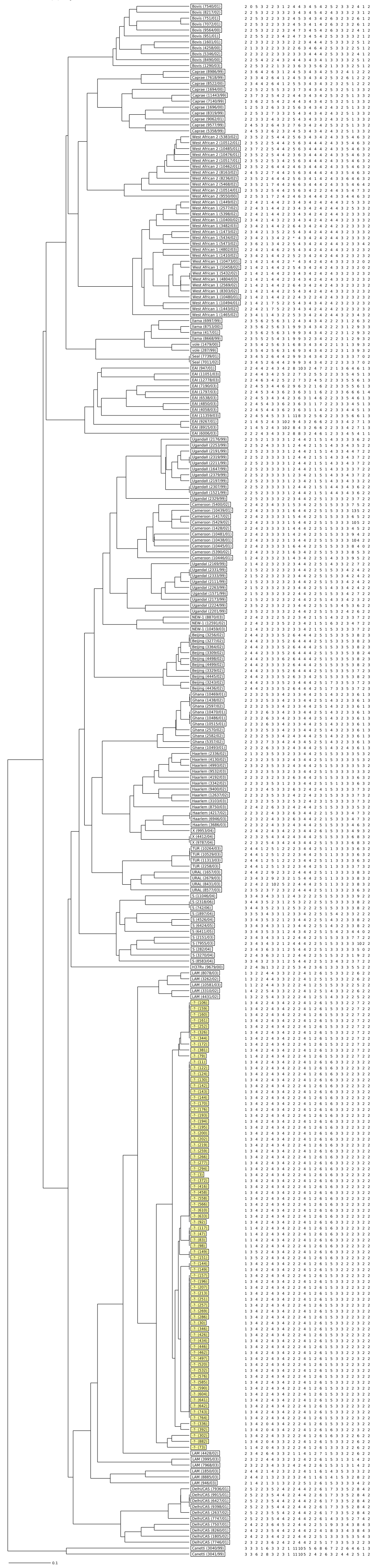

Supplement: Attachment — Submitted filename: miruvntr.pdf [file pone.0245084.s015.pdf]
