# Supplementary material for: First approach to the population structure of Mycobacterium tuberculosis complex in the indigenous population in Puerto Nariño-Amazonas, Colombia
Source: PLoS One. 2021 Jan 7;16(1):e0245084. doi: 10.1371/journal.pone.0245084 (PMC7790298; doi:10.1371/journal.pone.0245084)
Supplement: Attachment — Submitted filename: Response to Reviewers.docx [file pone.0245084.s016.docx]

To the attention of:

Dr. Igor Mokrousov

Academic Editor

PLOS ONE

Dear Dr. Mokrousov,

We would like to thank you as well as the reviewers for a constructive criticism of our paper. As you will see below, we have thoroughly revised the paper following a careful review of comments/observations made; the changes made are detailed below in order to meet the scientific and editorial needs underlined.

**Academic Editor**

***Query/Comment****: We note that [Figure(s) S1-11] in your submission contain [map/satellite] images which may be copyrighted. All PLOS content is published under the Creative Commons Attribution License (CC BY 4.0), which means that the manuscript, images, and Supporting Information files will be freely available online, and any third party is permitted to access, download, copy, distribute, and use these materials in any way, even commercially, with proper attribution. For these reasons, we cannot publish previously copyrighted maps or satellite images created using proprietary data, such as Google software (Google Maps, Street View, and Earth). For more information, see our copyright guidelines:* [*http://journals.plos.org/plosone/s/licenses-and-copyright*](http://journals.plos.org/plosone/s/licenses-and-copyright)*.*

**Authors**: We have used as maps an open access data service (OpenStreetMap®), licensed under the Open Data Commons Open Database License (ODbL) by the OpenStreetMap Foundation (OSMF), which is licensed under the Creative Commons Attribution-ShareAlike 2.0 license (CC BY-SA 2.0). The credits are clearly added to each map: “© OpenStreetMap contributors” and included the text: “Reprinted from OpenStreetMap under a CC BY-SA 2.0 license”

***Query/Comment****: Major concern. I would suggest that you do NOT build tree based on MIRU and spoligo together.*

*Rather it makes more sense to reanalyse your data in terms of clade assignment based only on 24-MIRU loci analysed together with 186 profiles in MIRU-VNTRplus. Otherwise addition of spoligo may create a confusion.*

**Authors**: We agree with your comment. Consequently, the dendrogram is made based only on 24-loci MIRU-VNTRs. Additionally, text was added in the manuscript emphasizing the fact that SIT392 might have been mislabeled as belonging to T family due to peculiar absence of spacers. Figures and table were also updated (with addition of “relabeled as LAM according to MIRU-VNTR analysis”) to highlight this fact.

***Query/Comment****: When I did this analysis (with your file you had sent me by email), it is clear that ALL your isolates belong to LAM and its RD115 sublineage. And this is unusual and interesting as half of LAM in Colombia belong to RD-Rio.*

**Authors**: By MIRU-VNTR profile, the isolates belonged to LAM sub-lineage RD115; a comment was made (Ln 422-426)

***Query/Comment****: I realise that it may be a challenge but there is a simple PCR-RFLP to detect LAM specific SNP in Rv0129c and it would be most advisable to run this test on your isolates.*

**Authors**: Due to the current situation, it was impossible for us to perform PCR-RFLP to detect LAM specific SNP in Rv0129c. We are sorry about it.

***Query/Comment****: Please provide excel file with all data*

**Authors**: An Excel file is now attached with all the required information.

***Query/Comment****: All figures (original files) are of low resolution.*

**Authors**: New figures were generated to improve resolution per recommendations of the journal.

**Reviewer #1**

***Query/Comment****: It may be worth reconsidering the relevance of Figure 3. A tree based only on spoligotyping data (single DR locus with non-independent characters/spacers) can lead to false clustering.*

**Authors**: We think that this figure (Fig 3) should be kept because it clearly shows a separation of strains from this study vs. other Colombian strains. Despite known limitation of spoligotyping data, some information from this technique could be taken into account, although further analyses (such as WGS) would be needed to better describe strains from this study. Text was modified accordingly.

***Query/Comment****: An interesting and important fact is that almost half of the samples were children. Perhaps it is worth clarifying is it a trend for this region? Is BCG vaccine given to children in this region?*

**Authors**: BCG immunization data will be published in another article that is in preparation, however patient information, plus a probable explanation of the amount of TB in children was added (Ln 319-323).

***Reviewer #2***

***Query/Comment****: In the description of the study sample, it is mentioned that 12 of the studied patients were identified by contact tracing. Was the genotype of these patients the same of their original contacts? I mean, was the strain of the patient identified by trace matching the strain of the patient that alerted that contact? If this were not the case, it would be warning of a potentially greater number of infected patients than those studied. It is an indirect measure of a high community transmission and of the effectiveness of screening; perhaps suggesting the need to extend screening to an unsuspected population.*

**Authors**: A brief explanation of cases detected by contact tracing was added. The percentage of patients with the same genotype as the index case is also noted, and discussed in (Ln 440-446).

***Query/Comment****: In the Figure 1 caption (dendrogram) there is an error in the description of the fields included, the fourth field starting from the left is the MIRU-VNTR pattern and not SITs based on SpolDB4, isn´t?*

**Authors**: The description fields were corrected. In addition, the dendrogram (figure 1) is now only based on 24-loci MIRU-VNTR data.

***Query/Comment****: In line 126 on page 6 when referring to DNA extraction, it should be indicated that it was extracted from the grown culture, because as it is written it could be confused with direct extraction from a clinical sample, and I understand that it is not the case. Perhaps this can be indicated in the previous section “DNA extraction”.*

**Authors**: Revised as suggested (Ln 121-122).

***Query/Comment****: In the population study, it is commented that 25.7% of the TB patients belonged to the Yagua ethnic group, but this group only accounts for 7.4% of the total population, it is, therefore, the ethnic group with the greatest affectation. What reason could there be for this situation? It does not seem linked to the communities where they reside, since this ethnic group is not particularly represented in the 2 populations with the highest number of tuberculosis cases, Puerto Nariño and San Francisco. Is there an epidemiological explanation or of any other kind that can explain it? It is very interesting and curious. Is there anything described in relation to this ethnic group that can justify this high incidence? Could they be, perhaps, the most exposed to a non-indigenous population?*

**Authors**: We do not have a concrete explanation for this observation, when looking at the demographic behavior, no significant difference was observed with the other 2 ethnic groups. However, one hypothesis is the movement of this ethnic group, which traveled frequently to Peru, where relatives live, this being perhaps a cause of their greater number of cases. This mobilization was observed during the field work, although it was not documented since no variables were obtained with regard to the mobility of the population. Further data and analysis are needed to better investigate this situation.

***Query/Comment****: With respect to patients with polyclonal/mixed infection:*

*a) How was the genotype of both strains involved deduced? Through the gel, it can only be interpreted that there is a locus with 2 alleles, but not which allele belongs to which strain. Were colonies picked to separate variants and genotyped to assign corresponding alleles? Otherwise, the allele combination may be different from that proposed in the manuscript and the genotypes, therefore, different. Was there a difference in intensity in the allele bands that allowed you to deduce which alleles were shared by the same strain? If one of the strains were represented in a lower proportion than the other, it is possible that the genotype can be deduced based on the intensities of the gel bands.*

*b) Has cross-contamination in the lab been ruled out? It is very strange that two patients have a mixed infection with the same 2 strains. Analysing the processing date of both strains, this laboratory contamination could be ruled out since if they had been processed at different times they could not have been mixed in the laboratory.*

**Authors**: Samples from patients with possible polyclonal infection were taken on 2 different days (May 25 and 26, 2016), so we consider that there is no cross contamination. However, the identification of double band in the MIRU-VNTR was after the field work, so it was not possible to take new samples from these patients, in addition, it was not possible to separate the colonies individually, these being the limitations as regards to polyclonal infection. Despite these limitations, we consider that it is important to mention the possibility of polyclonal infection in these patients, taking into account that the interaction time between them was very high since the 2 patients were in an emotional relationship.

***Query/Comment****: The paragraph explaining the cluster distribution in Figure 1 is confusing (199-206).*

*I understand that the final 8 clusters referred to are those named as 1-6 plus clusters a and b. If this is the case, in the last sentence, the "Additionally" (line 205) is left over, since they have already been included in the previous paragraph, it gives the feeling that they are 8 + 2. If I am wrong in my deduction, then it should be explained in more detail which 8 clusters the authors refer to, because it is not clear.*

**Authors**: Revised as suggested, the changes are made in figure 1.

***Query/Comment****: For the 6 cases of indigenous people that were identified as relapses (lines 272-273), was it possible to analyze whether they were reactivations or reinfections with a different strain? Were the strains from the first episodes available for genotyping by MIRU-VNTR? Due to the high rates of tuberculosis in this population, new exposure and therefore reinfection cannot be ruled out. It would be very interesting if you had made him comment on it.*

**Authors**: There is no data on previous genotyping in the study area, only information registered in the TB Program, so reinfection or reactivation cannot be determined (Ln 286-288).

***Query/Comment****: Regarding comparisons with other circulating strains, I´m lacking some comparisons mediated by MIRU-VNTR genotypes.*

*a) Have the study MIRU-VNTR genotypes been compared with those previously published by Cerezo and Realpe? Or with any other previous study that presents genotypes by MIRU-VNTR of circulating strains in Colombia or neighbouring countries? It is possible that some genotype was previously circulating and that once it was introduced into the indigenous population, it established itself as the dominant strain and subsequently evolved there endemically.*

*b) Did the strains with the LAM6-95 genotype found in Peru and Brazil (lines 327-334) share also the MIRU-VNTR type (1355) with the study strains?*

**Authors**:

**a)** It was compared with the 12 loci scheme, used by Cerezo in addition to the study carried out by Taype. Unfortunately the comparison is incomplete since 12 additional loci were not analyzed previously. However, with a scheme of 24 loci, the patterns appear as orphans.

**a)** According to the 12 loci scheme, the SIT95 and SIT1355 genotypes found in Peru share MIT201, as in the present study. However, as previously mentioned, the comparison is not complete (12 loci vs. 24 loci).

***Query/Comment****: It is strange as well as very interesting that the spoligotype breaks the genotypes formed by MIRU-VNTR when it is usually the contrary. It is true that the study population is very special, and this probably has something to do with it. I ask the authors as experts (out of curiosity) if they think it is likely that there is homoplasy between MIRU-VNTR genotypes, could it be?*

**Authors**: We did not comment on this point earlier not to be controversial, however, we do agree with the fact that homoplasy could exist between MIRU-VNTR genotypes (as shown in other studies). One could refer to a very recent study by Outhred et al. (Front. Public Health, 27 August 2020; <https://doi.org/10.3389/fpubh.2020.00455>) showing frequent homoplasy of MIRU repeats in modern *M. tuberculosis* lineages, arising through stochastic processes without natural selection. Nevertheless, our finding that the spoligotype in some cases can break MIRU-VNTR clusters remains worth reporting. Future studies using whole-genome single nucleotide polymorphism analysis could be useful to confirm this finding.
